# Factors Associated with Treatment Delay among Pulmonary Tuberculosis Patients in Public and Private Health Facilities in Addis Ababa, Ethiopia

**DOI:** 10.1155/2017/5120841

**Published:** 2017-02-27

**Authors:** Getinet Shewaseged Adenager, Fessahaye Alemseged, Henok Asefa, Amanuel Tesfay Gebremedhin

**Affiliations:** ^1^Department of Epidemiology and Biostatistics, Jimma University, Jimma, Ethiopia; ^2^Department of Population and Family Health, Jimma University, Jimma, Ethiopia

## Abstract

*Background*. Early detection and diagnosis of tuberculosis (TB) and the timely commencement of antituberculosis (anti-TB) treatment are the parts of efficient tuberculosis prevention and control program. Delay in the commencement of anti-TB treatment worsens the prognosis and increases the risk of death and the chance of transmission in the community and among health care workers.* Objective*. To assess tuberculosis treatment delay and associated factors among pulmonary TB patients in Addis Ababa, Ethiopia.* Methods*. A cross-sectional study was conducted in 10 public and 10 private health facilities that provide TB treatment. The data were collected from 425 newly registered pulmonary TB patients using pretested structured questionnaire from April to June 2012. Data were entered in EPI info version 3.5.1 and analyzed using SPSS version 16.0.* Findings*. The median durations of a patient, health care system, and total treatment delays were 17, 9, and 35 days, respectively. Overall 179 (42.1%), 233 (54.8%), and 262 (61.6%) of patients experienced patient delay, health care system delay, and total treatment delay, respectively. Distance more than 2.5 km from TB treatment health facility [AOR = 1.6, 95% CI (1.1–2.5)] and the presence of TB-associated stigma [AOR = 2.1, 95% CI (1.3, 3.4)] indicate higher odds of patient delay, whereas, being unemployed, patients with the hemoptysis symptom complain indicated lower odds of health care system delay [AOR = 0.41, 95% CI (0.24, 0.70)] and [AOR = 0.61 (0.39, 0.94)], respectively.* Conclusions*. A significant proportion of clients experienced patient and health care system delay. Thus, there is a need for designing and implementing appropriate strategies to decrease the delays. Efforts to reduce delays should give focus on integrating prevention programs such as active case detection and expanding access to TB care.

## 1. Introduction

According to WHO global tuberculosis report of 2012, tuberculosis (TB) affects the age group of 15 to 59 years old, which is an economically productive age group, and there were 8.7 million incident cases [range: 8.3 million–9.0 million] globally, equivalent to 125 cases per 100,000 population, and approximately 1.4 million died of TB in the year 2011 of whom 0.5 million were women. Most of the estimated number of cases in 2011 occurred in Asia (59%) and Africa (26%). The 22 high burden countries accounted for 81% of all estimated cases worldwide [1], where Ethiopia ranks seventh with an estimated incidence of all forms of TB of 378 new cases per 100,000 pop/year and 163 new smear-positive cases per 100,000 pop/year. The estimated prevalence of all forms of TB is 579/100,000 population, and smear-positive TB was 286/100,000 pop [1, 2]. The global target for TB control through full Directly Observed Treatment Short (DOTS) course expansion was the attainment of 70% case detection and attainment of 85% cure rate by 2005 [1, 3]. The Ethiopia DOTS detection rate was 36 percent, which is far behind the WHO's target of 70 percent detection [3].

Although different factors contribute to the low detection rate, studies conducted in different parts of the country indicated the patient delay in seeking treatment to be the main reason for high transmission and low detection rate [4–6]. The patient delay in seeking TB treatment together with health facility delay and referral delay leads to the treatment delay. Early diagnosis of the disease and prompt initiation of the treatment are essential for an effective tuberculosis control program. Delay in the initiation of tuberculosis treatment further increases the burden of tuberculosis by raising the probability of patients transmitting the infection and speeding up the emergence and transmission of multidrug-resistant (MDR) tuberculosis [1, 4–7]. Most transmissions occur from the onset of a cough to the initiation of treatment [8]. It is estimated that a patient with untreated smear-positive pulmonary TB may infect on average more than 10 persons per year and over 20 persons until death [1, 2]. Smear-negative pulmonary TB also plays a role in the spread of infection [1, 3]. Additionally, delay in initiation of tuberculosis treatment worsens the prognosis and increases the risk of death [1, 3, 4]. The treatment delay affects an individual, the community, and a country's health and economy [1]. In general, it is very difficult to quantify the crisis in country's health and economy, especially in the era of MDR-TB, from suffering and death of economically productive individuals from such preventable and treatable disease because of delayed initiation of treatment [1, 8].

In Ethiopia, studies on treatment delay among pulmonary patients are scarce. Likewise, recent information on treatment delay is highly limited especially in an urban setting, and unlike the previous studies, this study was conducted with pulmonary TB patients from both public and private facilities [4–6]. TB prevention and control is among the biggest public health challenges Ethiopia is facing. If the control of tuberculosis must succeed, magnitude and causes of the patient, health care system, and total treatment delays should further be investigated and curtailed. Hence, the purpose of this study was to investigate the magnitude and factors associated with the patient, health care system, and total treatment delays among pulmonary tuberculosis patients in Addis Ababa, Ethiopia.

## 2. Methods and Materials

### 2.1. Study Settings and Participants

The study was conducted in Addis Ababa city administration (capital of Ethiopia). The city has 10 subcities and a total population of 2.7 million. In the city, there were 24 public health centers and 25 private facilities providing TB treatment using DOTS program at the time of the study. A cross-sectional study was conducted in 10 public and 10 private DOTS-providing health facilities from April 3, 2012, to June 7, 2012. Both smear-positive and smear-negative pulmonary TB patients aged 15 years and above were included in the study. Relapse, failure, defaulters, and transfer in patients were excluded from the study since it is difficult to ascertain the date of the onset of symptoms and measure knowledge in these groups of patients. Additionally, patients who are severely ill were also excluded from the study.

### 2.2. Sample Size Determination

Single population proportion estimation formula was used with an assumption of 95% confidence interval, 5% significance level, 5% margin of error, 10% nonresponse rate, and 48% proportion of patient delay from a study conducted in Amhara region Ethiopia in 2005 [10]. Thus, the total sample size was 422.

### 2.3. Sampling Technique and Procedures

At the beginning of the study, 10 public health centers and 10 private health facilities were selected from 24 health centers and 25 private clinics, respectively. The selection of the health facilities was made from 10 subcities in Addis Ababa, and to ensure fair representation of each subcity, one public health center and one private health facility were selected randomly from the available public health centers and private health facilities in each subcity. Proportional allocation of patients according to the expected size of TB patients who seek care at each health facility was undertaken after reviewing previous years TB registries. Then, all pulmonary TB patients who were newly diagnosed and started anti-TB treatment during the study period were consecutively included in the study until the intended sample size was achieved.

### 2.4. Data Collection Instrument and Procedures

The data were collected from patients on the same day of initiation of anti-TB treatment by using an interviewer administered, locally translated (Amharic) questionnaire. The questionnaire was adopted with little modification from the WHO multicountry tuberculosis treatment delay survey and WHO ASSIST guideline [9, 11]. Data collectors were treatment providers working in the same health institutions. Adequate training was given for data collectors for 2 days on the content of the questionnaire and consent forms.

### 2.5. Data Quality Assurance

The questionnaire was pretested on 5% of the sample size in Addis Ababa in similar health facilities, which were not included in the sample and necessary modifications were made whenever needed. Frequent supervision of the data collection sites was made by the principal investigator and the assigned supervisors. Data collectors and supervisors checked the data for completeness and consistency daily. Completed quantitative data were entered into EPI info version 3.5.1 software. Then data were exported to SPSS version 16.0 and checked for inconsistencies and missing values by running frequencies and other data explorations. Inconsistencies and missing values were cleaned by checking the original questionnaire.

### 2.6. Data Analysis and Presentation

Description statistics such as mean, median, standard deviation, and percent were used to describe descriptive variables. Binary logistic regression was employed to investigate the association between independent variables and the outcome variables. Variables that showed significant association with the delays in the binary logistic regression at *P* value ≤ 0.25 were included in the multivariable logistic regression models to identify independent predictors to patient delay and health care system delay. In the multiple logistic regression models, the model association was considered at *P* value ≤ 0.05.

#### 2.6.1. Operational Definitions

*The Onset of Symptom.* It is the day when the patient first becomes aware of the symptom or symptoms.

*Patient Delay.* Patient delay is the time interval from the first onset of symptom to the first visit to health center or hospital or private clinics, and time longer than 21 days was considered indicative of patient delay [6, 7, 12, 13].

*Health Care System Delay.* Health care system delay is the time interval from the patient's first visit to the health center, hospital, or private clinic to the commencement of treatment, and time longer than 7 days is considered indicative of patient delay [6, 7, 12, 13].

**Figure 1:**
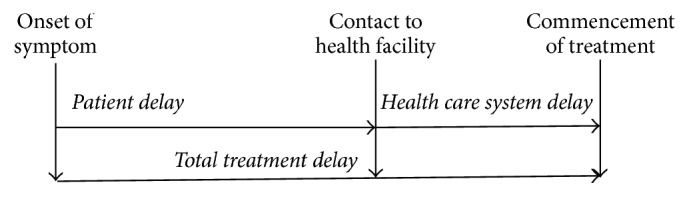
Components of different delay periods from the onset of symptom to the commencement of treatment. Modified from WHO diagnosis and treatment delay study [9].

*Total Treatment Delay.* Total treatment delay is the time interval from the first onset of symptom to the commencement of treatment or the sum of patient delay and health care system delay (Figure 1).

*Knowledge.* Variables measuring knowledge were recorded on a 3-point Likert scale of 10 questions (3 best and 1 worst). These included knowledge about the type of disease, its causes, curability, and type of antituberculosis drugs and duration of treatment. Patients who scored more than set average (50%) were considered knowledgeable and those who scored less than average were considered not knowledgeable [9].

*TB-Associated Stigma.* Variables measuring stigma were recorded on a 5-point Likert scale of 10 questions (5 the highest and 1 the lowest degree of stigma). These variables included feeling ashamed of having tuberculosis; having to hide tuberculosis diagnosis from others; delay to seek treatment due to fear of being diagnosed with TB; isolation due to tuberculosis; tuberculosis affecting the relationship with others; fear of TB/HIV coinfection. Patients who scored more than set average (50%) were considered to be having a high TB-associated stigma and those who scored less than average were considered to be having a low TB-associated stigma [9].

### 2.7. Ethical Consideration

Ethical approval was obtained from Jimma University Ethical Review Committee. Verbal consent from respondents was obtained before conducting the face-to-face interview. In order to maintain privacy and avoid the fear of stigmatization, health professionals working in TB units who already knew the serostatus of the clients were assigned to be data collectors. Patients were assured that the interview is private and confidential, their participation is voluntary, and their names will not be included in the questionnaire. Data collectors were provided proper advice for the respondents regarding any malpractice they have come across after the interview is completed.

## 3. Results

### 3.1. Patients Sociodemographic Characteristics

A total of 425 new smear-positive and smear-negative pulmonary tuberculosis (PTB) patients were consecutively included in the study with a response rate of 99.76%. Among the respondents, 237 (55.8%) were males, and 188 (44.2%) were females. The majority of the respondents accounting for 251 (59.1%) patients were in the age group of 15–34, and the mean age was 33.9 years, 320 (75.2%) were Christians, 188 (44.2%) were single, 176 (41.4%) were married, and 185 (43.5%) had primary education (Grades 1–8). Occupationally, 190 (44.7%) were employed. The majority of the patient's family income was between 15 and 45 USD (Table 1).

### 3.2. Clinical Characteristics and Health Seeking Behavior of Patients

There were 241 (56.7%) smear-positive and 184 (43.3%) smear-negative pulmonary TB patients. The primary symptoms that patients experienced during the onset of their illness included coughing 411 (96.7%), followed by loss of appetite 351 (82.6%) and fever 347 (81.6). A total of 218 (35%) of patients tried different forms of self-treatment at home and 334 (78.6%) patients were working in functional status before contacting formal health facility. In general, 218 (51.55) of patients first contacted public health facility, 302 (71.1%) patients were given drugs other than anti-TB drugs during their first visit to a health facility, 196 (46.1%) made at least two visits before diagnosis was made, and 263 (61.8) patients were diagnosed at public health facilities (Table 2).

### 3.3. Patient Delay

Overall 179 (42.1%) of patients did seek medical advice after 21 days (SD = 26.9) of the onset of their illness. The median patient delay was 17 days (IQR: 9–33) and the mean patient delay was 26 days (SD = 26.9). A total of 14 patients came on the same day of the onset of symptom. The minimum and maximum delays were 1 and 187 days, respectively. A total 382 patients (90%) came within two months and 15 patients (4.5%) came after 90 days.

Patients who traveled a distance more than 2.5 km to a TB treatment health facility were 1.6 times more likely to delay more than 21 days to contact a health facility when compared to patients who traveled a distance less than 2.5 Km (AOR = 1.6, 95% CI: (1.1, 2.5)).

Similarly, patients with high TB-associated stigma were 2.2 times more likely to delay more than 21 days to contact a health facility when compared with low TB-associated stigma (AOR = 2.1, 95% CI: [1.3, 3.4]) (Table 3).

### 3.4. Health Care System Delay

A total 233 (54.8%) pulmonary TB patients had a health care system delay of more than 7 days; of this, 136 patients (58.4%) first seek care at the public health facility and 97 (41.6) at the private health facility. The median health care system delay was 9 days (IQR: 3–29), and the mean was 19 days (SD = 22.9). The minimum and maximum health care system delays were 1 and 123 days, respectively. Exactly 101 (23.8%) patients delayed more than a month to start treatment, that is, 4 times the acceptable level of health care system delay (Table 4). In multiple variable logistic regression, patients with the symptom hemoptysis and being unemployed in occupation were less likely to experience health care system delay of more than 7 days once they contacted a health facility for their presenting symptom (AOR = 0.41, 95% CI (0.24, 0.70) and 0.61 (0.39, 0.94)), respectively (Table 4).

### 3.5. Total TB Treatment Delay

The median total treatment delay was 35 days (IQR: 19–63) and the mean was 45 days (SD = 35); the minimum and maximum total treatment delay were 2 days and 213 days, respectively. A total of 262 patients (61.6%) delayed more than 28 days before the initiation of treatment.

## 4. Discussion

This study shows that there is a significant delay from the onset of symptoms to the first contact to a health facility and from the first contact to initiation of anti-TB treatment for pulmonary tuberculosis patients in Addis Ababa.

Though the median patient delay of 17 days observed in this study is the acceptable level of patient delay [7, 13, 14], a significant proportion (41.2%) of TB patients experienced a patient delay of more than 21 days. This means that the substantial portion of TB patients in this study area did not go to health facilities early. Therefore, these groups of patients continued to serve as reservoirs of infection and will have continued to transmit the disease in the community until diagnosed and treated, which may have increased the burden of TB. The finding of this study is similar to other studies conducted in Ethiopia [4, 15] where a significant number of patients delayed contacting a health facility for more than 21 days. This study also showed a median patient delay lower than one in the findings of other studies conducted in Ethiopia [4, 6, 15] that showed a median patient delay of 28 to 56 days. This may be explained by the health care service access; these studies were conducted in the rural parts of Ethiopia where health seeking is believed to be low. Additionally, the median patient delay documented in this study was also lower than the one in the study conducted in Addis Ababa a decade ago, which reported a median patient delay of 60 days [5]. This can be explained by the TB prevention and control measures taken in the last 10 years. The median patient delay observed in our study is similar to ones in studies conducted in Vietnam [16], Hong Kong [17], and South India [18], where the reported median patient delay was between 14 and 21 days.

TB-associated stigma was one of the factors associated with a patient delay. Patients with a high level of TB-associated stigma were more likely to delay when compared to the patients with low TB-associated stigma. TB-associated stigma may have played an important role in hindering patients from seeking early health care due to fear of being diagnosed with TB. This finding is similar to discoveries in other studies conducted in Yemen, Syria, and Somalia [9]. Another variable that is associated with a longer patient delay was a distance greater than 2.5 km from TB treatment facility. This is because patients who came to a health facility from a distance more than 2.5 are from a relatively distant area from the health facility and this may create difficulties in accessing a health facility. Distance has proved to be an important factor for patient delay in studies conducted in Ethiopia [15] and South India [18]. Patient delay was not found significantly associated with age and sex in this study. Similar findings were reported in Ethiopia [6, 15], Gambia [19], Brazil [20], and Southern Taiwan [21]. However, studies conducted in Ethiopia [4], South Africa [22], South India [18], and Thailand [23] reported that females tend to seek health care lately compared to males. Similarly, older age was found significantly associated with patient delay in Ethiopia [15], South India [18], and Southern Taiwan [21].

It was observed that more than half of the patients experience a health care system delay. The median health care system delay of 9 days was documented in this study. Likewise, a health care system delay between 6 and 11 days was reported in studies conducted in Ethiopia [5] Nigeria [24], and Vietnam [16]. The median health care system delay documented in this study is lower than the findings of other studies conducted in different parts of Ethiopia [4, 6, 15]. These differences could be due to study area where the studies were conducted. Unlike this study, other studies are conducted in rural part of Ethiopia where health service coverage is low and available facilities may have limitations in the diagnosis of TB like lack of facilities for sputum examination, X-ray, and other supportive labs.

The median and magnitude of health care system delay observed in this study were unacceptably high. This may further increase the chance of transmission of TB in the community and to health professionals as well. Researches revealed that HIV or other immunesuppressing conditions often produce an atypical TB presentation that can weaken a patient's or their clinician's ability to detect TB, thus resulting in a diagnostic delay. In our study information was collected regarding comorbidities like HIV, even though the variable did not appear as being significantly associated with the delays.

Patients with the symptom hemoptysis were less likely to experience health care system delay more than 7 days. This may be explained by the fact that the symptom hemoptysis will easily remind health providers in TB diagnosis since it is a more serious symptom. Similar findings were reported in Indonesia and Nigeria [19, 25]. Unemployed patients were less likely to experience a health care system delay. This might be explained by assuming that unemployed people have enough time to revisit health facilities and to respect appointments given by health professionals for reevaluation.

Even though the association between health care system delay and the type of health facility first contacted disappeared after being controlled for other factors, it was found to be associated during binary logistic regression in this study. A similar finding is reported in West Ethiopia [4] and Uganda [26]. However, other studies reported contacting private clinic and health center in Ethiopia [6] and contacting the private facility in India [18] which were found associated with longer health care system delay. Hence, special attention should be paid for treatment seeking behavior of patients.

*Limitation of Study.* The current study has limitations that should be acknowledged. The recall bias might have been introduced as patients may not accurately estimate or remember the exact date of the onset of the first symptom and the date of the first visit made to a health facility. However, holidays and spiritual days were used as a reminder during the interview to minimize this bias. No information was collected to determine the length of time from the participants' first presentation of symptoms to the time of the interview. Information was not obtained for the time from the respondents first presentation of symptom to the time of interview. As there is no still agreed standard definition of delays in the treatment of TB, interpretations should be made cautiously.

## 5. Conclusions

In this study, it was observed that a significant proportion of patients delayed more than the acceptable level for patient and health care system delay. The study identified that a substantial proportion of TB patients in this study area did not get to health facilities early. Distance more than 2.5 km from TB treatment facility and high TB-associated stigma were found to be risk factors for prolonged patient delay. The hemoptysis symptom and unemployed occupational status were found to be protective factors not to experience longer health care system delay.

Based on findings of this study, it is the time to design active case detection strategies like TB screening of family members and community level suspect identification by using health extension workers to support the existing passive case detection strategy and decrease the length of patient delay. Moreover, it is important to increase the accessibility of TB diagnosis and treatment services by involving the remaining public health centers and private health facilities where TB services have not yet started. It is noted that distance between the patients' homes to a TB treatment facility had contributed to the delay in health care seeking. Additionally, health education sessions that emphasized the cause, the availability of treatment, and the curability of tuberculosis should be designed and provided to raise awareness and reduce the TB-associated stigma. Furthermore, as this is a cross-sectional study, future studies are essential that use stronger designs and can establish causality among different factors. Lastly, use of a standard definition of delay might permit for more suitable assessments.

## Figures and Tables

**Table 1:** Sociodemographic characteristics of pulmonary TB patients, Addis Ababa, Ethiopia, 2012.

Variables	*N* (%)
Sex	
Male	237 (55.7)
Female	188 (44.2)
Age in years	
15–24	119 (28)
25–34	132 (31)
35–44	88 (21)
45–54	49 (11.4)
55–64	22 (5.2)
65+	15 (3.5)
Educational status	
Unable to read and write	29 (6.8)
Able to read and write	39 (9.2)
Elementary (1–8)	185 (43.5)
Secondary (9–12)	104 (24.5)
Above secondary	68 (16)
Marital status	
Single	188 (44.3)
Married	176 (41.5)
Divorced	28 (6.6)
Widowed	33 (7.8)
Occupational status	
Employed	190 (44.7)
Unemployed	122 (28.7)
Housewife	42 (9.9)
Student	29 (6.8)
Merchant	34 (8)
Farmer	8 (1.87)
Religion	
Christian	320 (75.2)
Muslim	104 (24.4)
Family income	
<15 USD^a^	126 (29.6)
15–45 USD	210 (49.4)
>45 USD	89 (20.9)

^a^1  USD = 18.22 ETB.

**Table 2:** Clinical characteristics and health seeking behavior pulmonary TB patients in Addis Ababa, Ethiopia, 2012.

Patient characteristics	Number (%)
TB category	
Smear positive	241 (56.7)
Smear negative	184 (43.3)
Type of symptom	
Cough	411 (96.7)
Loss of appetite	351 (82.6)
Fever	347 (81.6)
Night sweating	286 (67.3)
Chest pain	263 (61.9)
Haemoptysis	168 (39.5)
Loss of weight	49 (11.5)
HIV status	
Positive	105 (24.7)
Negative	320 (75.3)
Contact history in the last 1 year	
Yes	96 (22.6)
No	329 (77.4)
Self-treatment	
Yes	149 (35.1)
No	276 (64.9)
Point of the first contact	
Public health facility	218 (51.3)
Private health facility	134 (31.5)
Drug outlets	45 (11.5)
Traditional healer	28 (6.5)
Severity of disease at the 1st contact	
Working	334 (78.6)
Ambulatory	86 (20.2)
Bed ridden	5 (1.2)
Services obtained at the 1st contact	
Advice only	6 (1.4)
Sputum is taken for examination	186 (43.8)
X-ray examination	199 (46.8)
Non-Anti TB drug given	302 (71.1)
Referred to a higher level	1 (0.2)
Total number of health-seeking encounters before diagnosis	
One	151 (35.5)
Two	196 (46.1)
More than two	78 (18.4)
Place of diagnosis	
Public health facility	263 (61.8)
Private health facility	162 (38.2)

**Table 3:** Factors associated with patient delay for treatment of PTB among pulmonary TB patients, Addis Ababa, 2012.

Variables	Patient delay		Unadjusted and adjusted OR	
	No delay	Delay	COR (95% CI)	AOR (95% CI)
Educational status				
Unable to read & write	12	17	1.2 (0.49, 3.0)	1.3 (0.48, 3.9)
Able to read & write	19	20	1.33 (0.61, 2.94)	0.55 (0.21, 1.5)
Elementary (1–8)	115	70	0.77 (0.44, 1.35)	0.93 (0.46, 1.9)
Secondary (9–12)	62	42	0.86 (0.46, 1.59)	0.77 (0.36, 1.7)
Above secondary	38	30	1	1
Marital status				
Single	121	67	1	1
Married	94	82	1.58 (1.0, 2.4)	1.0 (0.61, 1.6)
Widowed	14	14	1.81 (0.81, 4.01)	1.4 (0.56, 3.6)
Divorced	17	16	1.70 (0.80, 3.5)	1.7 (0.75, 3.8)
Occupational status				
Employed	109	81	1	1
Unemployed	74	48	0.87 (0.54, 1.39)	0.68 (0.40, 1.2)
Student	27	15	0.75 (0.37, 1.50)	0.82 (0.37, 1.8)
Housewife	15	14	1.26 (0.57, 2.75)	1.1 (0.46, 2.6)
Merchant	18	16	1.20 (0.57, 2.49)	1.2 (0.55, 2.6)
Farmer	3	5	2.24 (0.52, 9.66)	2.3 (0.48, 11.6)
Family size				
>5	27	31	1.7 (0.97, 2.9)	1.7 (0.93, 3.2)
≤5	219	148	1	1
Type of symptom				
Loss of weight				
Yes	36	13	**0.456** (0.23,0.88)^*∗*^	0.60 (0.28, 1.2)
No	210	166	1	1
Night sweating				
Yes	177	109	**0.60** (0.40,0.91)^*∗*^	0.70 (0.39, 1.24)
No	69	70	1	1
Loss of appetite				
Yes	212	139	0.49 (0.29,0.82)^*∗*^	0.72 (0.45, 1.2)
No	34	40	1	1
Point of first contact				
Traditional	10	18	3.11 (1.37,7.05)^*∗*^	0.38 (0.12, 1.2)
Drug out let	29	16	0.95 (0.49, 1.86)	0.78 (0.30, 2.0)
Private facility	69	65	1.63 (1.05,2.51)^*∗*^	0.59 (0.23, 1.5)
Public facility	138	80	1	1
Distance to health facility				
>2.5 km	147	76	1.4 (0.98, 2.0)	**1.6** (1.1,2.5)^*∗*^
≤2.5 km	278	103	1	**1**
TB associated stigma				
High stigma	127	129	2.4 (1.6,3.6)^*∗∗*^	**2.1** (1.3,3.4)^*∗*^
Low stigma	119	50	1	1
Knowledge				
Not knowledgeable	176	113	0.68 (0.45, 1.04)	1.2 (0.80, 2.0)
Knowledgeable	65	61	1	1

^*∗*^ *P* value < 0.05; ^*∗∗*^*P* value <0.001; COR: crude odds ratio; AOR: adjusted odds ratio.

**Table 4:** Factors associated with health care system delay for treatment of pulmonary TB among pulmonary TB patients, Addis Ababa, Ethiopia, 2012.

Variables	Health care system delay		Unadjusted and adjusted OR	
	No delay	Delay	COR (95% CI)	AOR (95% CI)
Sex				
Male	100	137	1	1
Female	92	96	0.76 (0.52, 1.12)	0.86 (0.56, 1.3)
Educational status				
Unable to read & write	13	16	1.16 (0.48, 2.78)	0.93 (0.35, 2.5)
Able to read and write	27	12	0.42 (0.18, 0.96)	0.89 (0.37, 2.1)
Elementary (1–8)	107	78	0.69 (0.39, 1.20)	1.1 (0.61, 2.1)
Secondary (9–12)	66	38	0.53 (0.28, 1.0)	1.2 (0.63, 2.4)
Above secondary	33	35	1	1
Occupational status				
Employed	76	114	1	1
Unemployed	74	48	**0.43** (0.27,0.68)^*∗∗*^	**0.41** (0.24,0.70)^*∗∗*^
Student	11	31	1.8 (0.89, 3.9)	2.1 (0.91, 4.6)
House wife	14	15	0.71 (0.32, 1.6)	0.55 (0.23, 1.3)
Merchant	15	19	0.84 (0.40, 1.8)	0.73 (0.34, 1.6)
Farmer	2	6	2 (0.39, 10.1)	1.8 (0.34, 10)
Marital status				
Single	92	96	1	1
Married	68	108	1.5 (1.0, 2.3)	1.5 (0.91, 2.4)
Widowed	17	11	0.62 (0.27, 1.4)	1.0 (0.41, 2.5)
Divorced	15	18	1.15 (0.55, 2.4)	1.2 (0.55, 2.8)
Family size				
≤5	171	196	1	1
>5	21	37	1.54 (0.87, 2.7)	1.7 (0.89, 3.2)
Type of symptom				
Loss of weight				
Yes	27	22	0.64 (0.35, 1.1)	0.85 (0.43, 1.7)
No	165	211	1	1
Haemoptysis				
Yes	88	80	**0.62** (0.41,0.92)^*∗*^	**0.61** (0.39,0.94)^*∗*^
No	104	153	1	
Loss of appetite				
Yes	167	184	**0.56** (0.33,0.95)^*∗*^	0.56 (0.30, 1.02)
No	25	49	1	1
Health facility first contacted				
Public health facility	123	136	1	1
Private health facility	69	97	1.3 (0.85, 1.8)	1.1 (0.70, 1.7)
TB associated stigma				
Low stigma	87	82	**1**	1
High stigma	105	151	**1.5** (1.03,2.2)^*∗*^	1.4 (0.90, 2.2)
Knowledge				
Not knowledgeable	48	78	1.5 (0.96, 2.3)	1.5 (0.93, 2.4)
Knowledgeable	138	151	1	

^*∗*^ *P* value < 0.05; ^*∗∗*^*P* value < 0.001, COR: Crude odds ratio, AOR: Adjusted odds ratio.

## References

[1] World Health Organization (WHO) (2011). *Global Tuberclosis Control*.

[2] World Health Organization (WHO) (2009). *Global Tuberculosis Control*.

[3] Federal Ministry of Health of Ethiopia (2008). *Tuberculosis/leprosy and TB/HIV Prevention and Control Programme Manual*.

[4] Wondimu T., W/Michael K., Kassahun W., Getachew S. (2007). Delay in initiating tuberculosis treatment and factors associated among pulmonary tuberculosis patients in East Wollega, Western Ethiopia. *Ethiopian Journal of Health Development*.

[5] Demissie M., Lindtjorn B., Berhane Y. (2002). Patient and health service delay in the diagnosis of pulmonary tuberculosis in Ethiopia. *BMC Public Health [Electronic Resource]*.

[6] Tegegen A., Yazachew M. (2009). Delay in tuberculosis treatment and associated factors in jimma zone Southwest Ethiopia. *Ethiopian Journal of Health Sciences*.

[7] Sreeramareddy C. T., Panduru K. V., Menten J., Van den Ende J. (2009). Time delays in diagnosis of pulmonary tuberculosis: a systematic review of literature. *BMC Infectious Diseases*.

[8] Centers for Disease Control and Prevention (2009). *Questions and Answers about Tuberculosis*.

[9] World Health Organization (WHO) (2006). *Diagnostic and Treatment Delay in Tuberculosis. An In-Depth Analysis of the Health-Seeking Behaviour of Patients and Health System Response in Seven Countries of the Eastern Mediterranean Region*.

[10] Storla D. G., Yimer S., Bjune G. A. (2008). A systematic review of delay in the diagnosis and treatment of tuberculosis. *BMC Public Health*.

[11] World Health Organization (WHO) (2010). *The Alcohol, Smoking and Substance Involvement Screening Test (ASSIST); Guidelines for Use in Primary Care*.

[12] Schneider D., McNabb S. J. N., Safaryan M., Davidyants V., Niazyan L., Orbelyan S. (2010). Reasons for delay in seeking care for tuberculosis, Republic of Armenia, 2006-2007. *Interdisciplinary Perspectives on Infectious Diseases*.

[13] Lambert M. L., Van Der Stuyft P. (2005). Editorial: delays to tuberculosis treatment: shall we continue to blame the victim?. *Tropical Medicine and International Health*.

[14] World Health Organization (WHO) (2003). *Treatment of Tuberculosis: Guidelines for National Programmes*.

[15] Yimer S., Bjune G., Alene G. (2005). Diagnostic and treatment delay among pulmonary tuberculosis patients in Ethiopia: a cross sectional study. *BMC Infectious Diseases*.

[16] Huong N. T., Vree M., Duong B. D. (2007). Delays in the diagnosis and treatment of tuberculosis patients in Vietnam: A Cross-Sectional Study. *BMC Public Health*.

[17] Leung E. C. C., Leung C. C., Tam C. M. (2007). Delayed presentation and treatment of newly diagnosed pulmonary tuberculosis patients in Hong Kong. *Hong Kong Medical Journal*.

[18] Rajeswari R., Chandrasekaran V., Suhadev M., Sivasubramaniam S., Sudha G., Renu G. (2002). Factors associated with patient and health system delays in the diagnosis of tuberculosis in South India. *International Journal of Tuberculosis and Lung Disease*.

[19] Lienhardt C., Rowley J., Manneh K. (2001). Factors affecting time delay to treatment in a tuberculosis control programme in a sub-Saharan African country: the experience of The Gambia. *International Journal of Tuberculosis and Lung Disease*.

[20] Dos Santos M. A. P. S., Albuquerque M. F. P. M., Ximenes R. A. A. (2005). Risk factors for treatment delay in pulmonary tuberculosis in Recife, Brazil. *BMC Public Health*.

[21] Chiang C.-Y., Chang C.-T., Chang R.-E., Li C.-T., Huang R.-M. (2005). Patient and health system delays in the diagnosis and treatment of tuberculosis in Southern Taiwan. *International Journal of Tuberculosis and Lung Disease*.

[22] Pronyk P. M., Makhubele M. B., Hargreaves J. R., Tollman S. M., Hausler H. P. (2001). Assessing health seeking behaviour among tuberculosis patients in rural South Africa. *International Journal of Tuberculosis and Lung Disease*.

[23] Rojpibulstit M., Kanjanakiritamrong J., Chongsuvivatwong V. (2006). Patient and health system delays in the diagnosis of tuberculosis in Southern Thailand after health care reform. *International Journal of Tuberculosis and Lung Disease*.

[24] Odusanya O. O., Babafemi J. O. (2004). Patterns of delays amongst pulmonary tuberculosis patients in Lagos, Nigeria. *BMC public health*.

[25] Kiwuwa M. S., Charles K., Harriet M. K. (2005). Patient and health service delay in pulmonary tuberculosis patients attending a referral hospital: A Cross-Sectional Study. *BMC Public Health*.

[26] Sendagire I., Van Der Loeff M. S., Mubiru M., Konde-Lule J., Cobelens F. (2010). Long delays and missed opportunities in diagnosing smear-positive pulmonary tuberculosis in Kampala, Uganda: a cross-sectional study. *PLoS ONE*.

